# Research on Monitoring Assistive Devices for Rehabilitation of Movement Disorders through Multi-Sensor Analysis Combined with Deep Learning

**DOI:** 10.3390/s24134273

**Published:** 2024-07-01

**Authors:** Zhenyu Xu, Zijing Wu, Linlin Wang, Ziyue Ma, Juan Deng, Hong Sha, Hong Wang

**Affiliations:** Institute of Biomedical Engineering, Chinese Academy of Medical Sciences, Tianjin 300192, China; s2023015013@pumc.edu.cn (Z.X.); wuzijing030208@163.com (Z.W.); wanglin_35@163.com (L.W.); zoeymm@yeah.net (Z.M.); pumcdj@126.com (J.D.); shahong2000@163.com (H.S.)

**Keywords:** rehabilitation assessment, auxiliary devices, accelerometer, six-axis force sensor, neural networks

## Abstract

This study aims to integrate a convolutional neural network (CNN) and the Random Forest Model into a rehabilitation assessment device to provide a comprehensive gait analysis in the evaluation of movement disorders to help physicians evaluate rehabilitation progress by distinguishing gait characteristics under different walking modes. Equipped with accelerometers and six-axis force sensors, the device monitors body symmetry and upper limb strength during rehabilitation. Data were collected from normal and abnormal walking groups. A knee joint limiter was applied to subjects to simulate different levels of movement disorders. Features were extracted from the collected data and analyzed using a CNN. The overall performance was scored with Random Forest Model weights. Significant differences in average acceleration values between the moderately abnormal (MA) and severely abnormal (SA) groups (without vehicle assistance) were observed (*p* < 0.05), whereas no significant differences were found between the MA with vehicle assistance (MA-V) and SA with vehicle assistance (SA-V) groups (*p* > 0.05). Force sensor data showed good concentration in the normal walking group and more scatter in the SA-V group. The CNN and Random Forest Model accurately recognized gait conditions, achieving average accuracies of 88.4% and 92.3%, respectively, proving that the method mentioned above provides more accurate gait evaluations for patients with movement disorders.

## 1. Introduction

Movement disorders, often referred to as extrapyramidal disorders, encompass a range of conditions characterized by involuntary movements, motor deficits, slowness and abnormal postures, often accompanied by cognitive or behavioral impairments and a marked decline in activities of daily living [1]. Rehabilitation medicine aims to promote the recovery of individuals with disabilities, enhance their quality of life, expedite the rehabilitation process, facilitate their return to independent living and alleviate the burden on families and society.

Motor rehabilitation assessment plays a crucial role in evaluating the effectiveness of rehabilitation interventions, with gait assessment being a primary modality. Early techniques, such as the footprint method, involve applying ink to animal paws and measuring gait patterns on paper but prove impractical for experimental use [2]. Motion capture systems, the gold standard for gait phase analysis, are expensive and complex [3]. With advancements in sensor technology, sensors like accelerometers have become popular for motion rehabilitation assessment due to their simplicity in terms of usage, affordable price and, above all, better integration when coupled with other sensors [4]. Motion capture systems are widely regarded as the gold standard for gait phase analysis. These systems typically employ high-precision optical sensors attached to human limbs and utilize high-speed cameras to capture motion, thereby acquiring the movement patterns of each joint. However, such systems are, too, expensive and complex to operate. Not to mention, such systems are prone to interference from environmental factors [5]. Over the past years, advancements in technology have made the manufacture of much more miniaturized yet equally accurate sensors possible [6]. Consequently, researchers are increasingly turning to sensor-based methods for sports rehabilitation assessment. Sensors such as accelerometers, plantar pressure sensors and electromyography sensors have proved to be effective when integrated into assessments [7,8]. These sensor-based systems are gaining prominence in gait assessment due to their simplicity, ease of integration and low cost. Additionally, computer vision-based gait analysis has shown great promise. For example, Nakano et al. [9] developed a 3D markerless motion capture technique using OpenPose with multiple synchronized video cameras, demonstrating that it could accurately reproduce movements with mean absolute errors below 30 mm in most cases. Binish Zahra et al. [10] compared marker-based and markerless motion capturing systems for human gait analysis, finding that machine learning approaches could achieve high accuracy in both person and activity recognition, with markerless systems offering a low-cost alternative.

Various studies have explored different sensor-based methods for gait analysis. Kotiadis et al. [11] designed an inertial gait phase detection system to replace heel and foot switches, testing four algorithms using accelerometers and gyroscopes to enhance gait analysis accuracy. Lee et al. [12] proposed a gait detection method using gyroscopes, accelerometers and magnetometers, accurately distinguishing normal walking from that of patients with spinal cord injuries. Niazmand et al. [13] integrated accelerometers in patients’ clothing to assess freezing of gait (FOG) during the assessment of Parkinson’s disease. Such a method, while achieving high accuracy and specificity, outperformed physician diagnoses. Ferrari et al. [14] compared gait data from accelerometers with video images, demonstrating less than a 5% stride length error. Samaniego et al. [15] successfully classified normal and abnormal gaits using accelerometers, highlighting their effectiveness. Accelerometers are also widely used in exoskeleton robot design to monitor and assist patient movement [16]. These studies highlight the versatility and effectiveness of various sensor-based systems in improving gait analysis and rehabilitation assessment.

In rehabilitation medicine, gait assessment is crucial for restoring patient function and monitoring treatment efficacy [17,18,19]. Traditional methods generally use a single sensor, especially accelerometers, to assess gait variability in patients, e.g., J. Barden et al. [20] used body-fixed sensors to assess gait variability, including gait symmetry, gait regularity and fractal structure for the elderly. However, for the rehabilitation assessment of patients with severe sports injuries and the self-balancing function assessment of lower limb exoskeleton robots, support from assistive devices is often required to assist patients in walking [21]. These situations present significant challenges because traditional assessment methods may not perform well in the presence of assistive devices or severe motor impairments.

To address these challenges, this study focuses on developing a novel rehabilitation assessment device that integrates accelerometers and six-axis force sensors. Unlike previous studies that have primarily used single-sensor systems, our device is designed to provide a more comprehensive evaluation by simultaneously monitoring gait and upper limb strength. The primary purpose of this study is to present a novel system designed to perform gait assessment for patients with lower limb motor impairments. This system aims to demonstrate its utility in accurately evaluating gait characteristics and providing reliable data for potential rehabilitation applications. By leveraging deep learning models to analyze data from multiple sensors, we aim to reduce the workload of therapists and provide a more objective evaluation tool. Specifically, accelerometers are used to assess gait, whereas force sensors measure changes in upper limb strength during the rehabilitation process. This dual-sensor approach enables a more detailed and accurate assessment of patient progress, particularly for those requiring assistive devices.

In summary, this study seeks to advance the field of rehabilitation assessment by introducing an innovative device that combines multiple sensors and advanced data analysis techniques. This approach addresses the limitations of traditional single-sensor methods and offers a more robust solution for monitoring and evaluating the rehabilitation progress of patients with movement disorders.

## 2. Materials and Methods

### 2.1. Auxiliary Vehicle

**Figure 1:**
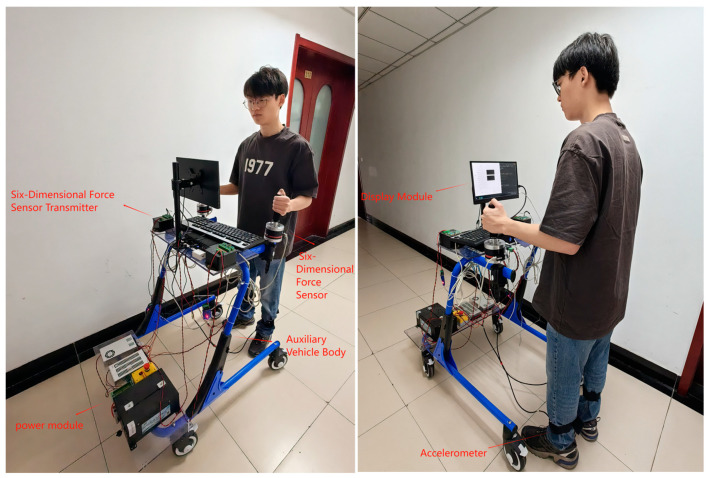
Auxiliary vehicle component module.

The primary structure of the auxiliary vehicle incorporates various components, including two accelerometers, two six-axis force sensors, two transmitters for the six-axis force sensors, the main unit, a display screen and a power module. This integration enables the gathering of the upper limb force and gait data during human rehabilitation assessments. The power module uses a matching voltage regulator to supply power to the sensors, main unit and display screen, effectively protecting the equipment and ensuring users’ safety during operation. The accelerometers, main unit and display screen are powered by a 12 V voltage regulator, and the six-axis force sensors and their corresponding multi-channel transmitters are powered by a 24 V voltage regulator. The configuration of the auxiliary vehicle is depicted in Figure 1. The height of the auxiliary vehicle is adjustable, making it suitable for individuals of different heights.

### 2.2. Accelerometer

**Figure 2:**
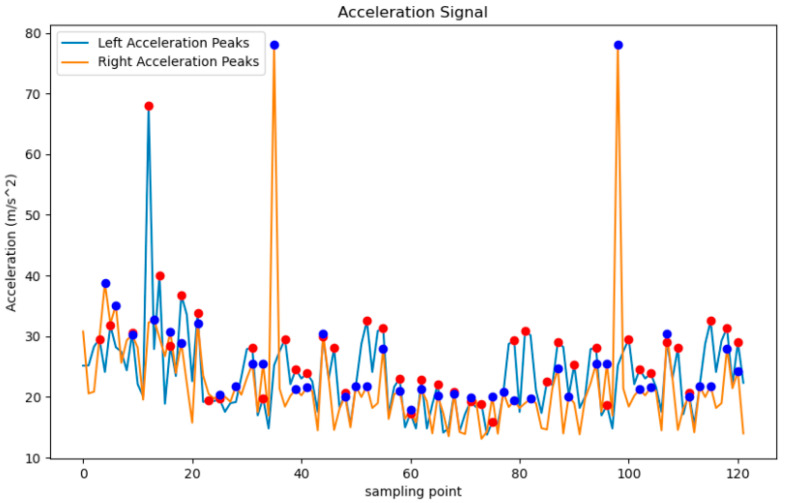
Accelerometer peak point extraction.

In this study, the HWT-605 accelerometer model was utilized, affixed to the participants’ ankles and secured with elastic bands. The collected acceleration data underwent processing steps such as filtering, denoising and other methods. Through the analysis of accelerometer data, key gait information such as gait cycle, average acceleration, peak acceleration and gait phases could be obtained to assess the patients’ walking gait. The HWT-605 accelerometer incorporates a tri-axial gyroscope and a tri-axial accelerometer. It features an RS485 interface and employs the Modbus-RTU communication protocol. In this study, the accelerometer was powered by a 12 V supply, with the sampling rate set to 60 Hz during the experiments. The accelerometer maintains good performance over prolonged use, with variations not exceeding 0.01 g. Its measurement range of ±16 g makes it suitable for scenarios with significant acceleration changes. Subsequently, peak values were extracted based on the characteristic that the value of a peak point exceeds that of its adjacent points on both sides, as depicted in Equation (1). The find_peaks function in the SciPy library of Python was employed for peak value extraction. However, noise points unrelated to the start and end points of the gait cycle may arise during the extraction process. To address this, parameters such as threshold and prominence were set in the find_peaks function. Consequently, index values of peak points in the original sequence data were obtained. Using an adaptive peak detection algorithm, the peak values of the accelerometer data were extracted (Figure 2), resulting in the indices of the peak points in the original sequence data. By subtracting the index values of each pair of adjacent peak points, the number of data points between two successive peaks was determined, as shown in Equation (1).

(1) data[i−1]<data[i] & data[i]>data[i+1]

where data[i] represents the value at index i in the data sequence.

The sensor sampling frequency f was set, and the gait cycle T was calculated using Equation (2), where T is the gait cycle duration, m is the number of data points between two peaks and f is the sampling frequency of the sensor. The peak acceleration data forms a sequence of peak coordinates corresponding to the peaks, and the efficacy of extracting gait cycle peak points is demonstrated in Figure 2. The red circles are the peak acceleration of the right hand, and the blue circles are the peak acceleration of the left hand.

(2) T=m/f

**Figure 3:**
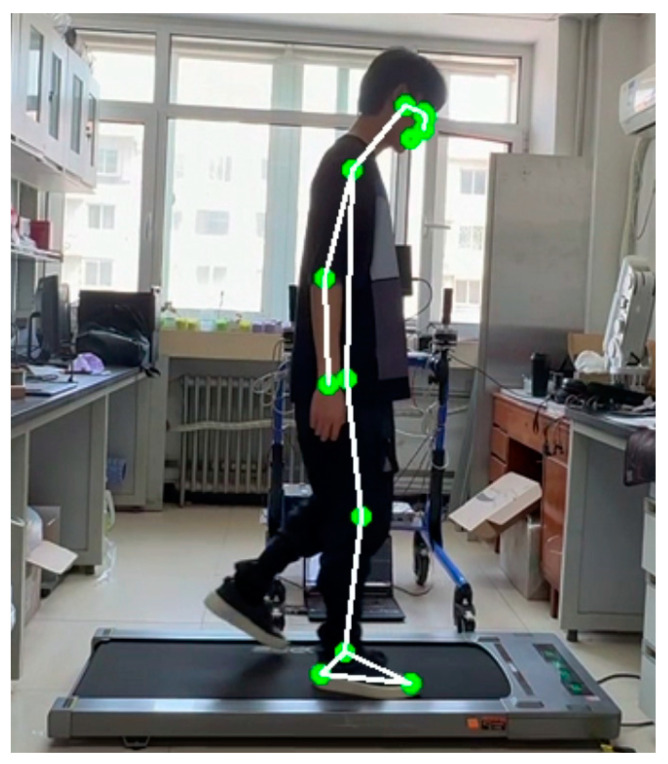
Key point recognition of motion capture system based on OpenPose.

In our study, we utilized a dynamic time–amplitude threshold peak extraction algorithm to identify peak points in the accelerometer’s gait waveform and to calculate the gait cycle. To validate the accuracy of our method, we compared these results with those obtained from an OpenPose-based motion capture system. The experiment involved a male subject, aged 24, walking on a treadmill at speeds of 1, 2, 3, 4 and 5 km/h. The subject wore the accelerometer correctly, and high-speed cameras recorded the walking process to capture key points, as depicted in Figure 3. The average cycle values measured by both systems were then compared.

### 2.3. Six-Axis Force Sensor

**Figure 4:**
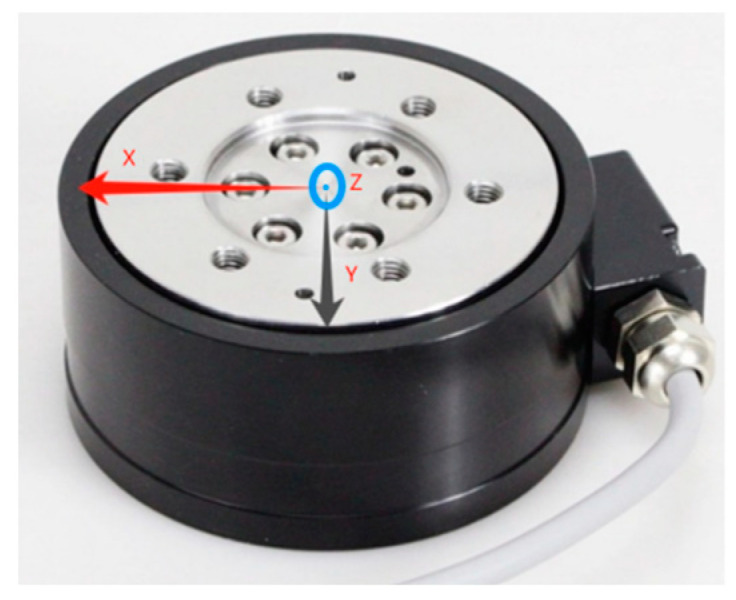
Six-axis force sensor.

Six-axis force sensors are capable of measuring changes in upper limb force during rehabilitation assessments, encompassing three-dimensional forces (Fx, Fy, Fz) and three-dimensional moments (Mx, My, Mz). During the measurement process, the mean and standard deviation of forces in the X, Y and Z directions for both the left and right sides, along with the resultant force, were recorded. The resultant force represents the combined effect of the forces in the X, Y and Z directions and is computed using Equation (3). This capability allows for the analysis of changes in upper limb assistive force during different stages of rehabilitation. Each axis direction of these sensors has a force measurement range from 0 to 1000 N and a moment measurement range of 30 Nm, which is suitable for monitoring the upper limb assistive forces of most people. The sampling rate was set to 60 Hz, with a supply voltage of 24 V. The six-axis force sensors utilized an RS485 interface and Modbus-RTU communication protocol. The orientations of the six-axis force sensors during measurement are depicted in Figure 4. A flange, designed using SolidWorks 2022, was used to attach the sensors to the base securely. This flange interfaces with both the upper and lower surfaces of the force sensor and is securely affixed to the auxiliary vehicle.

(3) $$Resultant\;Force(F)=\sqrt{{Fx}^{2}+{Fy}^{2}+{Fz}^{2}}$$

### 2.4. Method

This study recruited 50 graduate students as participants, with an average age of 23.196 years (SD = 2.017), an average height of 168.319 cm (SD = 7.295) and an average weight of 64.125 kg (SD = 12.427). The experiment included two primary groups: a normal walking (NW) group and an abnormal walking group. The abnormal walking group was further divided into a moderately abnormal (MA) group, a moderately abnormal with vehicle assistance (MA-V) group, a severely abnormal (SA) group and a severely abnormal with vehicle assistance (SA-V) group. The grouping details are shown in Table 1.

To assess the performance of the equipment, we conducted experiments to compare gait characteristics under different simulated injury conditions and to validate the effectiveness of the assistive device.

**Figure 5:**
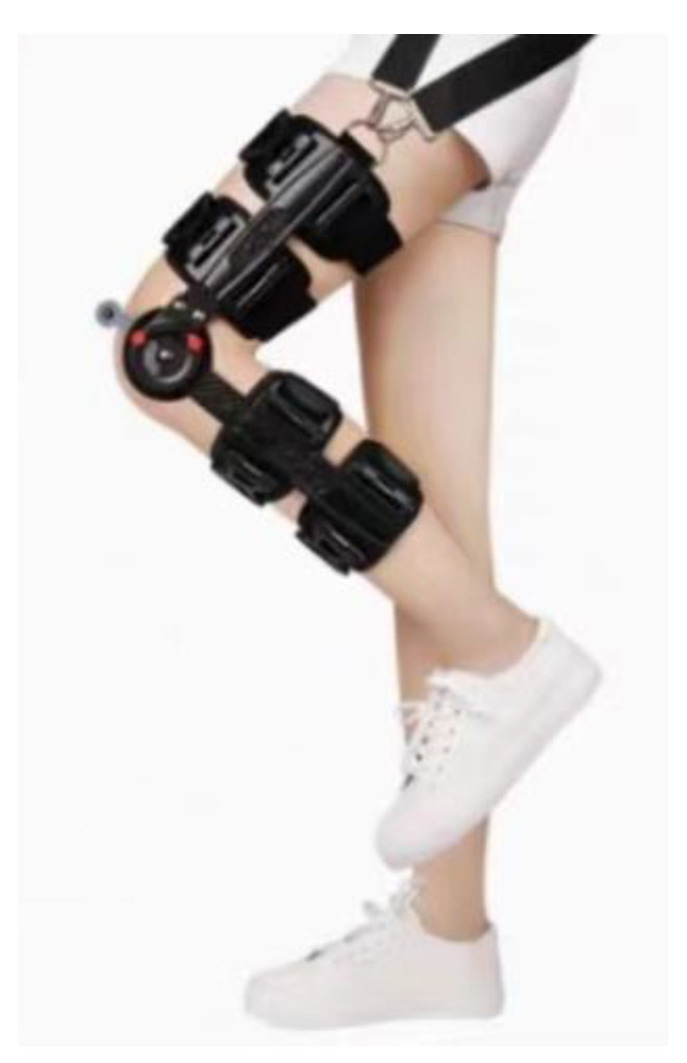
Knee immobilization device.

The experiment was conducted in the corridor of an experimental office building. Participants were fitted with a knee joint fixation device, which was adjusted to different ranges of motion angles to simulate varying degrees of knee injuries, as shown in Figure 5. Each participant walked 300 steps under each of the following conditions: normal walking (NW), moderately abnormal walking (MA), moderately abnormal walking with vehicle assistance (MA-V), severely abnormal walking (SA) and severely abnormal walking with vehicle assistance (SA-V). The conditions were not randomly assigned; each participant experienced all conditions in a predetermined sequence to ensure consistency and minimize variability due to order effects.

Data collection included accelerometer readings from both legs and six-axis force sensor readings from both hands. Each group comprised 50 samples, resulting in a total of 300 steps per participant per condition. Participants performed 2–3 trial walks to adapt to the knee joint fixation device before data collection commenced. During the experiment, participants stood in a relaxed posture in the designated testing area before receiving instructions to walk.

The primary instruments used on the equipment during the experiment included two accelerometers and two six-axis force sensors, with accelerometers capturing three-dimensional acceleration data from both legs and force sensors measuring three-dimensional forces and resultant forces exerted by the upper limbs. This setup allowed us to comprehensively analyze the gait patterns and validate the system’s performance under different simulated injury conditions.

Data from multiple sensors were analyzed using deep learning models to quantitatively assess the rehabilitation progress of patients with lower limb motor impairments. Key metrics included gait symmetry and upper limb auxiliary force.

**Figure 6:**
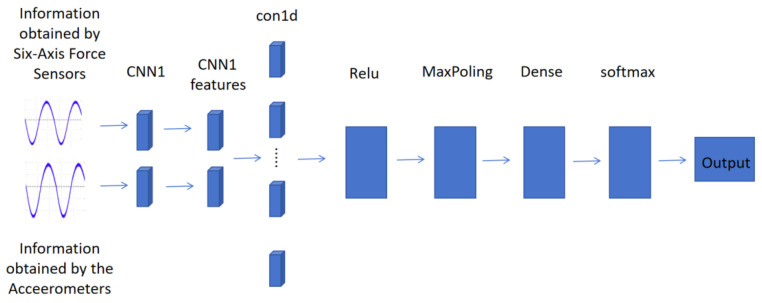
Schematic of CNN model.

In this study, we proposed a deep learning model based on the fusion of multi-sensor data, the overall architecture of which is illustrated in Figure 6. The sensors used included two six-axis force sensors held by the participants’ left and right hands and two accelerometers attached to the participants’ left and right ankles using elastic bands. These sensors provided comprehensive data on both upper limb forces and lower limb movements, respectively. These features were then merged and processed through a one-dimensional convolutional layer with 64 convolutional kernels, utilizing the ReLU activation function. After that, the output of the convolutional layer underwent max-pooling, and the outputs of the convolutional and pooling layers were linked to the subsequent fully connected layer. The output layer consisted of three neurons corresponding to three categories: healthy individuals, moderately abnormal patients and severely abnormal patients. The softmax function was employed for the classification task.

Furthermore, important features extracted from the sensor data were used as input to a decision tree forest model to evaluate its effectiveness in classifying different gait conditions. Implemented using the Scikit-learn library in Python 3.9.18 [22], the Random Forest Model was employed to classify rehabilitation data accurately. This ensemble learning method, composed of multiple decision trees, enhances classification accuracy by combining their predictions through voting or averaging. The purpose of using this model is to demonstrate its potential in accurately identifying gait abnormalities, which can be critical for assessing and improving gait rehabilitation strategies. The dataset was divided into training and testing sets, with 80% of the data used for training the model and 20% reserved for evaluating its performance.

The primary purpose of this study is to present a system and demonstrate its utility in performing gait assessment for patients with gait impairments. This study aims to compare normal and abnormal gait patterns using data collected from both upper and lower limb sensors and to validate the effectiveness of the proposed system in accurately classifying different gait conditions through the use of machine learning models, including a Random Forest Model. Additionally, this study seeks to provide a quantitative assessment tool that can aid in improving gait rehabilitation strategies for patients with lower limb motor impairments. By integrating sensor data and employing deep learning techniques, this study enhances the understanding of gait abnormalities and contributes to more effective rehabilitation strategies.

### 2.5. Statistical Analysis

The data collected during the experiment were saved in CSV files or stored in Excel format. Statistical analysis was conducted using SPSS 22.0 software, with statistical significance set at *p* < 0.05. Continuous variables, including gait cycle, peak acceleration and mean acceleration for both legs from the accelerometer data, as well as the mean force, standard deviation of the force and peak force for both hands from the six-axis force sensor data, were tested for normality using the Graphpad prism 10.1.2 test. For normally distributed variables, paired *t*-tests were used to compare gait cycle and the mean acceleration between the moderately abnormal walking (MA) and severely abnormal walking (SA) groups, as well as between the moderately abnormal walking with vehicle assistance (MA-V) and severely abnormal walking with vehicle assistance (SA-V) groups. An analysis of variance (ANOVA) was employed to compare the peak acceleration and standard deviation of acceleration across all groups (MA, MA-V, SA, SA-V). For force sensor data, paired t-tests compared the mean force, standard deviation of the force and peak force between the MA-V and SA-V groups, as well as between the MA and SA groups, and an ANOVA assessed the dispersion of force across all groups. The results indicated no significant differences in gait cycle and mean acceleration between the MA-V and SA-V groups but significant differences between the MA and SA groups. The ANOVA revealed significant differences in peak acceleration and standard deviation between the MA-V and SA-V groups. The force sensor data analysis showed a higher dispersion of force in the SA-V group, with significant differences in mean force, standard deviation and peak force between hands, indicating greater reliance on the unaffected upper limb in the SA-V group. These findings underscore the importance of integrating accelerometer and force sensor data for a comprehensive evaluation of gait asymmetry in patients with different stages of motor impairment.

### 2.6. Scoring

In this study, a Qt graphical user interface was developed, incorporating functions for data collection, real-time data display and comprehensive evaluation. Following the selection of evaluation criteria, the data underwent normalization using the extreme value method. Features were then extracted utilizing convolutional neural networks (CNNs), and accuracy was predicted. These features encompassed the acceleration of the left and right legs and the six-axis force of the left and right hands, as well as the absolute differences in acceleration between the left and right directions. Additionally, features included the standard deviations, means and peaks of the six-axis force differences. The classification algorithm utilized the Random Forest Model from the Scikit-learn toolbox in Python. Model performance was evaluated using the test set, and accuracy was assessed by calculating the model’s accuracy rate.

## 3. Results

### 3.1. Accelerometer

**Figure 7:**
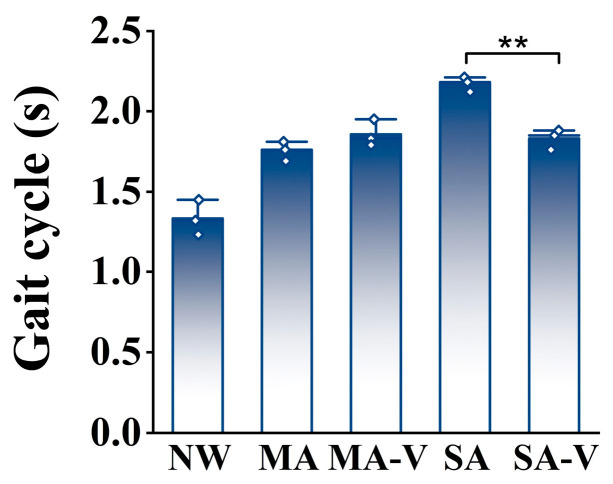
Normal and abnormal walking gait cycles. ** (0.001 ≤ *p* < 0.01).

During the experiment, the average gait cycle duration for a normal gait was 1.31 ± 0.0757 s, whereas for an abnormal gait, it was approximately 1.98 ± 0.2374 s, indicating an increase of 0.67 s relative to a normal gait. There was no significant difference (*p* > 0.05) in the gait cycle duration between the moderately injured group (MA), the MA group walking with the aid of the vehicle (MA-V) and the severely injured group (SA). However, the gait cycle of the MA-V group was slightly higher than that of the MA group, possibly due to a restricted arm swing when patients used the vehicle. In contrast, there was a significant difference (*p* < 0.05) in the gait cycle duration between the severely injured group (SA) and the SA group walking with the aid of the vehicle (SA-V), with the gait cycle of the SA-V group being significantly lower than that of the SA group, indicating that the use of the vehicle effectively assisted patients with severely abnormal gaits. There was a significant difference (*p* < 0.05) between the MA group and the SA group. No significant difference (*p* > 0.05) was observed between the MA-V and SA-V groups when both utilized the vehicle. Figure 7 presents the average gait cycle durations under different walking modes. The experiment revealed that the normal gait cycle duration was significantly shorter than that of abnormal gait conditions. The use of the auxiliary vehicle (MA-V and SA-V) influenced the gait cycle, particularly in severely abnormal conditions, where the vehicle significantly reduced the gait cycle duration. This demonstrates the vehicle’s potential effectiveness in assisting patients with severe gait impairments. Note: Statistical significance is indicated by * (0.01 ≤ p < 0.05) and ** (0.001 ≤ *p* < 0.01).

Throughout the experiment, the right leg was simulated as impaired. The results indicated that, for all abnormal groups, the average acceleration and peak acceleration of the left leg exceeded those of the right leg, with significant differences (*p* < 0.05) observed in the peak acceleration between the left and right legs. Patients primarily relied on rapid swinging of the left leg to support their body during walking. 

In the validation analysis, as shown in Table 2, the results showed no significant statistical differences (*p* > 0.05) in the average gait cycles measured by the motion capture system and the accelerometer across different walking speeds. The maximum average error between the two systems was 0.015 s, demonstrating the high accuracy of the gait cycles obtained using the adaptive peak detection algorithm.

**Figure 8:**
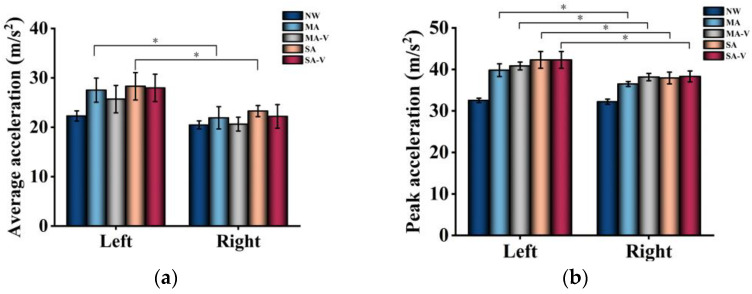
Mean and peak values of left and right leg accelerometers. (**a**) Average acceleration. (**b**) Peak acceleration. * (0.01 ≤ *p* < 0.05).

Figure 8a depicts the average acceleration of the left and right legs during measurement. The results revealed significant differences (*p* < 0.05) in average acceleration between the left and right legs within the MA and SA groups. Specifically, the standard deviation of acceleration for the left leg in the MA group was 2.44, whereas for the right leg, it was 2.25. Conversely, for the SA group, the standard deviation of acceleration for the left leg was 2.77, and for the right leg, it was 1.13. Among the abnormal groups, the standard deviation of acceleration for the left leg exceeded that of the right leg, indicating poor symmetry between the legs during walking and an unstable gait. No significant differences (*p* > 0.05) were observed in the average acceleration of the left and right legs in the MA-V and SA-V groups.

Figure 8b illustrates the peak acceleration values obtained during the analysis. The accelerometer sensors were positioned on the participants’ ankles and secured with elastic bands, as mentioned earlier in Section 2. Each sensor recorded the acceleration data at a sampling rate of 60 Hz. The analysis of 600 steps provided comprehensive insights into the gait patterns of the participants, validating the effectiveness of the proposed system. Note: Statistical significance is indicated by * (0.01 ≤ *p* < 0.05) and ** (0.001 ≤ *p* < 0.01).

### 3.2. Six-Axis Force Sensor

**Figure 9:**
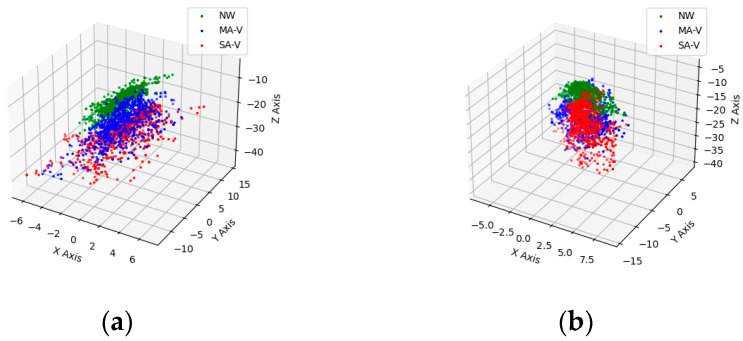
Scatterplot of left- and right-hand forces. (**a**) Scatter plot of left-hand force. (**b**) Scatter plot of right-hand force.

Six-axis force sensors played a pivotal role in capturing changes in upper limb force throughout the rehabilitation assessment process. During measurement, the mean and standard deviation of forces in the X, Y and Z directions for both the left and right sides, along with the resultant force, were recorded, as outlined in Table 3. The findings revealed that, during abnormal walking scenarios, the mean force (absolute value) exerted by the left hand notably exceeded that exerted by the right hand, particularly in the Z-axis direction. For instance, in the MA-V group, the left-hand force surpassed that of the right hand by 4.23 N, whereas in the SA-V group, it was 9.87 N higher, indicating a tendency for the body’s center of gravity to lean toward the healthy side during walking. Consequently, greater force was necessitated on the left upper limb to sustain the patient’s gait. Table 3 depicts the mean and standard deviation of the resultant force. Notably, the standard deviation of the resultant force on the left side in the NW group stood at 0.732 N, whereas in the MA-V and SA-V groups, it escalated to 2.524 N and 3.207 N, respectively. Additionally, the force scatter plot of the left and right hands, illustrated in Figure 9, showcased that the scatter plot of forces in the SA-V group exhibited the highest dispersion, whereas the NW group displayed better concentration. This observation suggests that patients with movement impairments experience significant directional variations in forces during walking to maintain balance. The discrepancy in force between the MA-V group and the NW group on the right hand was insignificant, possibly attributed to patients with moderate movement disorders sustaining body balance by exerting force on the healthy side’s upper limb, thereby resulting in minimal differences in force between the affected side and normal walking. Leveraging the concentration of the scatter plot of upper limb force can aid in classifying patients with varying degrees of movement impairments, thereby facilitating the monitoring of rehabilitation effectiveness among patients assisted with the vehicle.

### 3.3. Model Training

**Figure 10:**
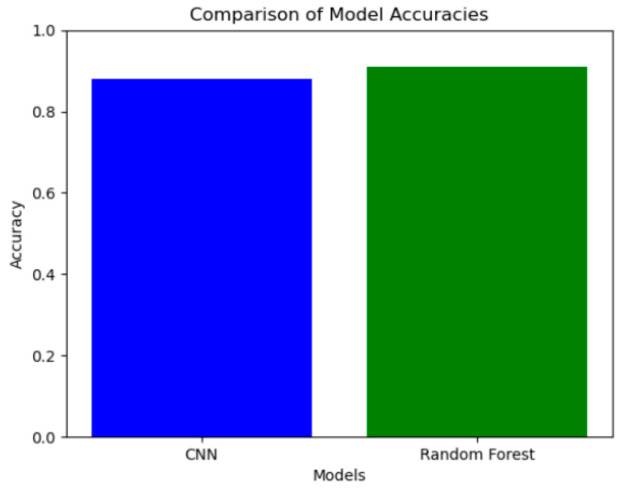
Model accuracy.

**Figure 11:**
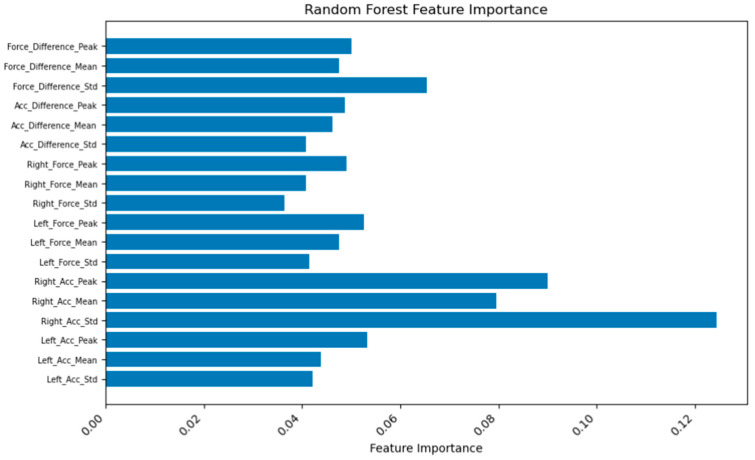
Weight of important features in the classification task.

Our convolutional neural network (CNN) effectively learned features from both accelerometer and six-axis force sensor data. The model achieved an accuracy of 88.4% on the test set, demonstrating its capability to classify rehabilitation data accurately (Figure 10). The Random Forest Model, which identified important features from the sensor data, achieved a higher accuracy of 92.3%. Figure 11 presents the distribution of feature weights in the classification task, indicating the significance of each feature in the model’s predictions. The model’s superior performance underscores its effectiveness in classifying different gait conditions and its potential in assessing rehabilitation progress.

## 4. Discussion

The primary objective of this study was to develop an auxiliary vehicle equipped with sensors to evaluate the rehabilitation outcomes of movement disorders. Our findings revealed that relying solely on accelerometer data for gait analysis during rehabilitation assessments with an auxiliary vehicle proved insufficient in discerning between varying degrees of movement impairment and detecting asymmetry between the left and right limbs. Therefore, we introduced measurements of upper limb force to complement the analysis. The clustering and dispersion patterns observed in the scatter plots of the upper limb force effectively distinguished between different stages of movement rehabilitation, offering additional parameter insights for rehabilitation assessment [23]. The results showed significant differences in gait parameters between the affected and unaffected limbs under different walking conditions. Specifically, this study found that the average and peak accelerations of the left (unaffected) leg significantly exceeded those of the right (affected) leg in all abnormal groups (*p* < 0.05). This highlights that patients predominantly relied on the unaffected leg for support during walking. The gait cycle computed by identifying peak values in the accelerometer data in our study showed similar trends to findings by Jakob et al. [24] using a motion capture system. True validation of our system requires a more rigorous approach, including comparisons with gold standard methods such as motion capture systems or force plates across diverse data collection scenarios. Our experiment comparing accelerometer results with those from the OpenPose-based motion capture system demonstrated that the two methods produced statistically similar gait cycle measurements across various walking speeds. The maximum average error of 0.015 s between the two systems indicates the high accuracy of our adaptive peak detection algorithm. These findings support the reliability of our accelerometer-based method for gait analysis. Future studies should focus on comprehensive validation by collecting data from a wider range of patients and incorporating various data treatment methods. By doing so, we can ensure that our accelerometer-based gait analysis method is robust, accurate and reliable across different conditions and patient populations. This thorough validation process is essential to confirm the effectiveness of our system in clinical settings. This rigorous approach ensures that our accelerometer-based gait analysis method is accurate and reliable across different conditions and patient populations. Past research in motor rehabilitation assessment has focused on gait analysis. Accelerometer-based gait analysis by H. Matsumoto et al. [25] recently provided a sensitive measure of functional decline and gait variability for patients suffering from various motor dysfunctions, and this motor rehabilitation assessment did not take into account changes in upper limb strength. The significant difference in gait cycle durations between the MA and SA groups and the lack of a significant difference between the MA-V and SA-V groups indicate that the auxiliary vehicle may help in reducing gait cycle disparities caused by varying levels of injury severity. This finding demonstrates the vehicle’s potential in providing consistent support across different levels of gait impairment. To ensure the reliability of these results, further validation is necessary. Repeated experiments and cross-validation with independent datasets can help confirm the accuracy of our measurements. 

The observed data show that, in the SA group, the standard deviation of acceleration for the left leg was significantly higher than that for the right leg, highlighting a lack of symmetry and indicating an unstable gait. This poor symmetry is a common characteristic in patients with severe injuries. However, in the MA-V and SA-V groups, the use of the vehicle appeared to mitigate these discrepancies, as no significant differences were observed in the average acceleration between the left and right legs. This suggests that the vehicle might help in reducing asymmetry in gait, thereby providing more balanced support for both legs.

Additionally, incorporating error analysis and comparing our findings with those obtained through other established methods can provide a comprehensive validation of our approach. The accelerometer and force sensor data add robustness to our findings by offering multiple perspectives on the patients’ gait characteristics. These multi-sensor analyses contribute to a more holistic understanding of rehabilitation progress and device effectiveness. 

Some researchers have also used features extracted from sensor data to build models to classify patients with motor dysfunction and healthy individuals. Oh et al. [26] used EEG signals to construct a convolutional neural network (CNN) model to detect Parkinson’s disease with an accuracy of 88.25%. Zheng et al. [27] used wearable sensors to collect 95 gait parameters by building a support vector machine (SVM) to classify Parkinson’s disease patients and healthy controls with an accuracy of 96.67% and specificity of 96.55%.

Furthermore, leveraging convolutional neural networks (CNNs) and Random Forest Models, we extracted features and constructed a model to classify patients with movement disorders and healthy individuals. Our deep learning model, structured with a convolutional neural network, effectively learned features from both accelerometer and six-axis force sensor data, achieving an accuracy of 88.4%. Additionally, the Random Forest Model, which verified the important features, achieved a higher accuracy of 92.3%, demonstrating superior performance due to the richer dataset and advanced analytical methods employed. Our results demonstrate that the scoring system, combining a CNN and Random Forest Model, achieved an impressive accuracy of up to 92.3% in detecting the true gait conditions of patients, effectively discriminating gait characteristics across different walking modes.

Historically, studies on movement rehabilitation assessment have predominantly centered on gait analysis, often overlooking changes in upper limb strength [28,29]. Our study, however, revealed significant differences in upper limb strength between the left and right hands during abnormal walking. Patients with walking difficulties tended to redistribute their body weight onto the healthy side to facilitate walking [30]. For individuals with severe movement impairments, the assistance of devices may be necessary to complete walking, underscoring the relevance of our study for reassessing these patients’ retraining progress.

It is imperative to acknowledge the limitations of our study, notably the constraints imposed by experimental conditions, which precluded the collection of real walking data from actual patients. The abnormal gait observed in our experiment was simulated by healthy individuals wearing knee joint fixation devices. Future research endeavors should prioritize collecting authentic data from patients exhibiting varying degrees of movement impairment and explore the integration of more sophisticated models to enhance the accuracy of evaluating the rehabilitation outcomes of movement disorders.

## 5. Conclusions

This study introduced an auxiliary vehicle outfitted with accelerometers and six-axis force sensors, enabling effective monitoring of changes in upper limb force and lower limb acceleration during walking, alongside the extraction of pertinent gait features through multi-sensor coupled analysis. By innovatively amalgamating rehabilitation and assessment functionalities, our research presents a novel avenue for movement rehabilitation assessment. The utilization of the auxiliary vehicle offers practicality for evaluating the rehabilitation progress of severely impaired patients unable to ambulate independently, particularly when reliant on assistive devices for mobility. By integrating a convolutional neural network (CNN) and the Random Forest Model, this study’s rehabilitation assessment system precisely evaluates the gait of patients with movement disorders, thereby furnishing robust support for advancements in the realm of rehabilitation medicine.

## Figures and Tables

**Table 1:** Experimental groups.

Experimental Group	Knee Joint Fixation Angle
Normal Walking (NW)	No knee joint fixation device
Moderately Abnormal (MA)	0–30°
Moderately Abnormal with Vehicle (MA-V)	0–30°
Severely Abnormal (SA)	No knee movement
Severely Abnormal with Vehicle (SA-V)	No knee movement

**Table 2:** Gait cycle calculations at different speeds (seconds).

	1	2	3	4	5
Motion Capture System	1.779 ± 0.114	1.306 ± 0.084	1.117 ± 0.040	0.986 ± 0.037	0.814 ± 0.034
Accelerometer	1.787 ± 0.107	1.291 ± 0.060	1.128 ± 0.039	0.981 ± 0.046	0.802 ± 0.031
Error	0.008	0.015	0.011	0.005	0.012
*p*-value	0.629	0.388	0.425	0.230	0.600

**Table 3:** Variation in upper limb assistive forces evaluated by six-axis force sensors.

Groups	Side	Axial	Force (N)	Resultant Force (N)	Peak Force (N)
NW	left	X	−0.811 ± 0.463	20.861 ± 0.732	31.899
		Y	2.573 ± 0.583		
		Z	−20.742 ± 1.696		
	right	X	−0.608 ± 0.488	17.304 ± 0.758	26.318
		Y	1.124 ± 0.614		
		Z	−16.634 ± 1.728		
MA-V	left	X	−2.084 ± 1.412	25.680 ± 2.524	38.646
		Y	3.505 ± 1.688		
		Z	−25.291 ± 5.145		
	right	X	0.128 ± 1.712	21.398 ± 0.979	35.327
		Y	0.874 ± 1.774		
		Z	−21.061 ± 4.041		
SA-V	left	X	−2.265 ± 1.136	28.795 ± 3.207	41.698
		Y	3.574 ± 1.422		
		Z	−28.114 ± 8.292		
	right	X	1.274 ± 2.307	18.928 ± 1.204	40.631
		Y	2.715 ± 2.711		
		Z	−18.612 ± 4.875		

## Data Availability

The data used for this study cannot be shared publicly, so supporting data are not available.
